# Advancements in Minimally Invasive Techniques in Pediatric Dentistry: A Review

**DOI:** 10.7759/cureus.76929

**Published:** 2025-01-05

**Authors:** Abdulrahman M Alqahtani, Yazed D Alshihri, Abdulaziz E Alhumaid, Mohammed M Al Nafaie, Abdullatif A Alnaim

**Affiliations:** 1 General Dentistry, Private Dental Clinic, Khobar, SAU; 2 Dentistry, Imam Abdulrahman Bin Faisal University, Dammam, SAU; 3 Dentistry, Qassim University, Buraidah, SAU; 4 Pediatric Dentistry, King Fahd Military Medical Complex (KFMMC), Dammam, SAU; 5 Dental Public Health, University College London, London, GBR

**Keywords:** 3d printing, atraumatic restorative treatment (art), bioactive materials, caries management, laser-assisted therapies, minimally invasive dentistry (mid), noninvasive techniques, pediatric dentistry, resin infiltration, silver diamine fluoride (sdf)

## Abstract

Minimally invasive dentistry (MID) has revolutionized pediatric dental care by emphasizing the preservation of healthy tooth structures, reducing treatment-related trauma, and improving patient compliance. This narrative review explores advancements in MID techniques, including silver diamine fluoride (SDF), resin infiltration, atraumatic restorative treatment (ART), bioactive materials, laser-assisted therapies, and three-dimensional (3D) printing technologies. These approaches prioritize early diagnosis, prevention, and conservative management, aligning with patient-centered and sustainable practices. SDF demonstrates high efficacy in arresting caries progression but presents esthetic challenges due to discoloration. Resin infiltration provides esthetic and noninvasive treatment for white spot lesions, while ART offers cost-effective and child-friendly caries management in resource-limited settings. Bioactive materials support tissue regeneration, and laser technologies enable precise and painless procedures, although their adoption is limited by high costs and training requirements. Emerging tools, such as artificial intelligence and 3D printing, enhance diagnostic accuracy and treatment precision. Despite challenges related to cost, operator training, and infrastructure, MID techniques continue to evolve, offering promising solutions for pediatric dental care. Future research should focus on optimizing materials, improving accessibility, and integrating digital technologies to broaden the impact of minimally invasive approaches. This review highlights MID’s transformative role in improving oral health outcomes and ensuring sustainable, patient-focused care for children.

## Introduction and background

Minimally invasive dentistry (MID) has emerged as a transformative approach in modern dental care, emphasizing conservation of healthy tooth structures while effectively treating dental diseases [1]. It shifts away from the traditional "extension for prevention" approach, which frequently requires the excessive removal of healthy tooth structures, and instead emphasizes early diagnosis, prevention, and minimally invasive interventions [2]. MID aligns with global efforts to promote sustainability, patient-centered care, and improved clinical efficiency [3]. In pediatric dentistry, MID holds special significance due to its emphasis on minimizing psychological trauma, improving treatment outcomes, and reducing dental anxiety [4].

Pediatric patients present unique challenges in dental care due to their developmental stages, fear of dental procedures, and limited ability to cooperate during treatments [5]. Traditional invasive treatments, such as drilling and filling, often exacerbate dental anxiety and result in avoidance behaviors, contributing to the progression of untreated dental diseases [6]. MID aims to address these challenges by replacing conventional drilling methods with noninvasive approaches, preserving primary dentition until natural exfoliation, and focusing on preventive strategies [7]. As primary teeth play a critical role in speech development, chewing efficiency, and guiding permanent teeth eruption, preserving them is vital for both functional and psychological health [8].

The foundation of MID lies in its ability to combine early diagnosis, preventive measures, and conservative treatments. Techniques such as silver diamine fluoride (SDF), resin infiltration, atraumatic restorative treatment (ART), and laser-assisted therapies offer effective and less traumatic solutions for managing dental caries [3]. SDF, in particular, has demonstrated exceptional success in arresting caries progression without the need for anesthesia or drilling, making it suitable for children with high caries risk, special needs, or dental anxiety [4].

Studies have shown that SDF not only arrests caries but also promotes remineralization through its fluoride content, providing long-term protection against further decay [3]. Despite its advantages, the black staining caused by SDF is often an esthetic concern for parents and older children [5]. Strategies such as the silver-modified atraumatic restorative treatment (SMART) technique, which combines SDF with esthetic glass ionomer restorations, have been developed to overcome this limitation [2].

Resin infiltration is another pivotal technique in MID, targeting noncavitated enamel lesions [6]. Unlike traditional restorative methods, resin infiltration uses a low-viscosity resin that penetrates and seals the lesion, halting progression and preserving the structure [7]. This technique is particularly beneficial for managing white spot lesions post-orthodontic treatment, as it eliminates the need for drilling and provides highly esthetic results [8]. Additionally, it enhances compliance among pediatric patients by eliminating pain and reducing treatment time [5].

ART has gained prominence as a cost-effective and field-friendly technique, especially in low-resource settings [7]. It involves the removal of caries using hand instruments and restoration with fluoride-releasing glass ionomer cement [6]. ART does not require complex equipment, making it ideal for school-based and rural health programs [3]. Its simplicity and effectiveness in caries management have led to its integration into public health initiatives worldwide [8].

Advances in laser-assisted therapies have further expanded the scope of MID, providing precise and painless options for caries removal and pulp therapies [7]. Laser systems allow for minimal thermal damage, reduced bleeding, and faster healing, making them well-suited for pediatric patients who may fear traditional rotary instruments [8]. However, the high costs and training requirements associated with laser equipment pose barriers to widespread adoption [5].

The COVID-19 pandemic accelerated the adoption of minimally invasive techniques, as they aligned with infection control protocols and reduced aerosol generation [6]. Procedures such as SDF application, ART, and resin infiltration became preferred options during the pandemic, highlighting their utility in maintaining oral health while ensuring patient and practitioner safety [4]. This experience has emphasized the importance of integrating MID practices into long-term dental care strategies [3].

Modern bioactive materials have also contributed to the success of MID by enhancing remineralization and tissue regeneration [5]. Materials such as nanohydroxyapatite, calcium silicate cement, and bioactive glass interact with the surrounding tooth structure, releasing ions that strengthen enamel and prevent lesion progression [7]. These materials align with MID principles by actively participating in the healing process rather than merely filling cavities [8].

Artificial intelligence (AI) has further improved MID by enabling early diagnosis and precision treatment planning [4]. AI-powered tools analyze radiographs, detect lesions, and assess caries risk with high accuracy, supporting evidence-based decision-making [3]. Combined with digital imaging, AI enhances the effectiveness of MID techniques by allowing early interventions and minimizing invasive procedures [5].

Despite its many advantages, MID faces certain challenges. The esthetic concerns associated with SDF, the high cost of lasers, and the need for operator skill and moisture control for resin infiltration limit widespread adoption [6]. Efforts to overcome these barriers include training programs, improved materials, and low-cost technologies that can extend MID applications to underserved populations [8].

Future directions for MID involve advancements in regenerative therapies, three-dimensional (3D) printing technologies, and AI-guided diagnostics [7]. These developments aim to make treatments more precise, efficient, and accessible [8]. Continued research is essential to validate the long-term effectiveness of MID approaches, further cementing their role as the gold standard for pediatric dental care [3].

## Review

Materials and methods

This narrative review focuses on exploring the latest advancements in minimally invasive techniques in pediatric dentistry. It highlights diagnostic tools, clinical applications, and treatment modalities such as SDF, resin infiltration, ART, and laser-assisted therapies. The review synthesizes data from peer-reviewed journals, systematic reviews, and clinical studies published between 2018 and 2024.

Study Design

This study adopts a narrative approach, summarizing existing literature related to minimally invasive techniques in pediatric dentistry. A comprehensive evaluation of diagnostic methods, restorative treatments, and emerging technologies was performed to identify their effectiveness, challenges, and future directions.

Search Strategy

A targeted literature search was conducted in online databases, including PubMed, Google Scholar, and Consensus, using keywords such as the following: "Minimally invasive dentistry in pediatric patients," "Silver diamine fluoride in children," "Resin infiltration techniques," "Atraumatic restorative treatment in pediatric dentistry," and 'Laser-assisted dental therapies."

Inclusion and Exclusion Criteria

Inclusion and exclusion criteria were defined to ensure the selection of high-quality, relevant studies. Table 1 provides a summary of the criteria applied during the literature review.

**Table 1:** Inclusion and exclusion criteria

Inclusion criteria	Exclusion criteria
Peer-reviewed studies (2018-2024)	Non-English publications
Focus on minimally invasive pediatric dentistry	Studies focusing on adult populations
Clinical trials, systematic reviews, meta-analyses	Case reports with insufficient sample sizes
Articles evaluating diagnostic and treatment techniques	Anecdotal evidence or opinions

Data Extraction and Analysis

Data were extracted on diagnostic techniques, treatment approaches, outcomes, and limitations. Quantitative and qualitative comparisons were made to evaluate the efficacy and feasibility of each approach. Trends and gaps in research were identified to propose directions for future studies.

Review

Silver Diamine Fluoride (SDF)

SDF has emerged as one of the most significant advancements in MID. It is a topical agent combining the antimicrobial properties of silver with the remineralizing effects of fluoride, providing a dual-action approach to arrest and prevent caries progression [3]. Its simplicity, affordability, and noninvasive application make it particularly suitable for pediatric patients with high caries risk, special needs, or dental anxiety [4].

SDF functions by forming a protective layer of silver protein complexes that inhibit bacterial activity, while fluoride ions promote enamel remineralization [5]. Multiple studies have confirmed its efficacy in halting caries progression, making it especially valuable for treating young children and individuals with limited access to dental care [2]. Beyond its therapeutic benefits, SDF has demonstrated preventive effects when applied to sound surfaces, reducing the incidence of new lesions [6]. Table 2 summarizes the benefits and limitations of SDF in caries management.

**Table 2:** Benefits and limitations of silver diamine fluoride (SDF)

Benefits	Limitations
Noninvasive and painless application [3]	Causes black staining on carious lesions [5]
Highly effective for caries arrest and prevention [4]	Requires reapplication for long-term efficacy [2]
Cost-effective and ideal for public health programs [6]	Esthetic concerns may affect patient acceptance [5]
Suitable for special needs and uncooperative children [3]	Contraindicated in patients with silver allergies [4]

The black discoloration caused by SDF remains its most cited limitation, especially for anterior teeth where esthetics are a primary concern. Additionally, SDF requires repeated applications to maintain its effectiveness, which may affect compliance in some cases. It can also cause contact irritations to soft tissues if not applied carefully. Furthermore, contraindications, such as silver allergies, must be considered before application [2,5]. To address this issue, techniques such as the SMART combine SDF application with glass ionomer cement (GIC) restorations, preserving functionality while masking discoloration [2].

The application of SDF has gained traction globally, especially in school-based programs and community health initiatives. Research has demonstrated its long-term effectiveness when combined with periodic reapplications, suggesting that it can significantly reduce the burden of untreated dental caries in underserved populations [7]. Future advancements aim to improve its esthetic properties without compromising its antimicrobial efficacy [8].

Resin Infiltration

Resin infiltration represents a breakthrough in MID by effectively addressing early-stage caries and enamel defects without drilling or extensive tooth preparation [2]. This technique involves infiltrating low-viscosity resins into porous enamel structures to seal and strengthen lesions, halting their progression [3]. Its esthetic benefits make it particularly suitable for treating post-orthodontic white spot lesions and noncavitated caries in pediatric patients [4]. The ability to preserve natural tooth structures while minimizing discomfort aligns perfectly with pediatric dentistry principles [6].

The process of resin infiltration typically involves pretreatment with hydrochloric acid for 15-60 seconds to open enamel pores, enhancing resin penetration and effectiveness [9,10]. Alternative pretreatment approaches, such as phosphoric acid etching and abrasive surface conditioning, have also demonstrated success in increasing resin penetration while improving safety for young children [3]. These modifications enhance the applicability of resin infiltration in pediatric dentistry [4].

Clinical studies highlight the effectiveness of resin infiltration in arresting noncavitated carious lesions, with success rates ranging from 80% to 90% in slowing lesion progression [6]. Its ability to mask white spot lesions has shown positive esthetic results, particularly for patients undergoing orthodontic treatments [7]. A systematic review and meta-analysis confirmed the high efficacy of resin infiltration in managing proximal caries lesions, further validating its use in preventive pediatric care [8].

In addition to treating carious lesions, resin infiltration has been explored for managing developmental enamel defects such as fluorosis and hypomineralization [9]. Studies have demonstrated that resin infiltration can reduce lesion visibility while preserving enamel integrity, making it an effective option for children with esthetic concerns [10]. Its ease of application and ability to avoid invasive procedures make it particularly useful for patients with behavioral challenges or dental anxiety [11].

Recent innovations in resin materials have further expanded its potential. Bioactive resins incorporating fluoride, calcium, or antibacterial agents have shown promise in promoting remineralization and resisting secondary caries [12]. Experimental formulations are being tested to enhance resin durability, penetration depth, and long-term stability [13]. These advancements are expected to improve treatment outcomes and broaden the scope of resin infiltration [13,14].

Despite its numerous advantages, resin infiltration has some limitations, such as the need for precise moisture control, higher costs, and limited effectiveness in cavitated lesions. These limitations, along with accessibility concerns, are summarized in Table 3.

**Table 3:** Benefits and limitations of resin infiltration

Benefits	Limitations
Noninvasive, preserving enamel structure [2]	Requires precise moisture control [3]
Effective for white spots and noncavitated lesions [4]	Limited application for cavitated lesions [6]
Esthetically pleasing and avoids anesthesia [7]	Technique-sensitive and time-consuming [8]
Promotes remineralization with bioactive resins [12]	High material cost may limit accessibility [13]

Future developments in resin infiltration focus on improving the material properties to enhance penetration depth, durability, and resistance to secondary caries [12]. Bioactive resins and antibacterial formulations are promising advancements that may further expand their applications in pediatric dentistry [14].

Atraumatic Restorative Treatment (ART)

ART is a cornerstone of MID, offering effective and affordable care for managing dental caries in pediatric patients [6]. Developed as a technique to address barriers to dental care in resource-limited settings, ART utilizes hand instruments for caries removal and fluoride-releasing GIC for restoration [14]. Its adaptability to various clinical scenarios makes it a valuable tool for preventive and restorative care [15].

ART aligns with the principles of MID by emphasizing maximum preservation of healthy tooth structures while providing cost-effective and pain-free treatments [2]. It is particularly effective for treating single-surface lesions, with survival rates of over 90% at 12 months and nearly 77% at 36 months, as reported in long-term studies [6]. The use of GIC further supports remineralization through fluoride release, preventing recurrent caries [16].

Several studies have explored ART’s effectiveness compared to other techniques. For example, a 36-month trial comparing ART and the Hall Technique found ART to be equally effective for primary molars but better suited for noncooperative children due to its simplicity and reduced anxiety [17]. Moreover, ART has shown high acceptance among patients and parents, reinforcing its role as a child-friendly alternative [18].

Recent advancements in ART focus on combining it with other minimally invasive approaches, such as SDF and SMART techniques, to enhance clinical outcomes [3]. This hybrid approach improves ART’s longevity and provides additional antimicrobial benefits [15]. Table 4 highlights the benefits and limitations of ART as a minimally invasive approach for managing dental caries.

**Table 4:** Benefits and limitations of atraumatic restorative treatment (ART)

Benefits	Limitations
Minimally invasive and child-friendly [6]	Limited application for deep or multisurface cavities [16]
No anesthesia or rotary instruments required [15]	Requires skilled operators for precision [17]
Fluoride release supports remineralization [3]	Durability concerns in larger cavities [18]
Cost-effective and suitable for community programs [16]	Requires periodic follow-ups for maintenance [15]

Although ART is highly effective, its limitations include reduced success rates for multisurface lesions and larger cavities, where alternative approaches may be needed [16]. Operator skills and material handling are also critical to achieving optimal results [17]. Recent developments in bioactive materials and antimicrobial coatings aim to enhance ART’s durability and performance [18].

Future directions for ART focus on integrating bioactive glass ionomers and antibacterial formulations to improve restorative outcomes [15]. Combining ART with emerging techniques, such as laser-assisted therapies, may further expand its scope and ensure greater success rates in pediatric dentistry [15].

Bioactive Materials in Minimally Invasive Pediatric Dentistry

Bioactive materials have revolutionized minimally invasive pediatric dentistry by promoting natural remineralization, tissue repair, and long-term durability [5]. These materials interact with tooth structures through ion exchange, supporting regeneration and improving bond strength, making them ideal for minimally invasive approaches [19]. Their application in pediatric dentistry ranges from direct restorations and pulp capping to indirect restorations and endodontic therapies [20].

The primary advantage of bioactive materials lies in their ability to release calcium, phosphate, and fluoride ions, fostering enamel and dentin remineralization [21]. Biodentine, a widely used bioactive material, demonstrates excellent biocompatibility, bioactivity, and adaptability, making it suitable for restorative and endodontic treatments in children [22]. Similarly, bioactive glasses are effective for regenerating enamel and dentin structures due to their ability to form hydroxyapatite-like layers [23].

Bioactive materials, including tri-/dicalcium silicate cement, release calcium and phosphate ions, supporting remineralization, pulp vitality, and periapical tissue repair [24]. These materials also exhibit antibacterial properties, contributing to improved long-term outcomes [21]. Bioactive composites, incorporating nanotechnology, have further enhanced mechanical strength and durability, allowing for broader applications in pediatric dentistry [25].

In clinical practice, bioactive materials are used for procedures such as indirect pulp capping, cavity lining, and sealing early-stage carious lesions [20]. They reduce the need for extensive cavity preparations, aligning with the principles of MID [19]. Additionally, their ability to stimulate reparative dentin formation makes them ideal for managing deep caries and pulp exposures [22]. Table 5 summarizes the benefits and limitations of bioactive materials in restorative dentistry.

**Table 5:** Benefits and limitations of bioactive materials

Benefits	Limitations
Promotes natural remineralization and tissue repair [5]	Higher cost compared to conventional materials [22]
Releases fluoride, calcium, and phosphate ions [21]	Requires precise handling and moisture control [24]
Supports pulp vitality and prevents secondary caries [19]	Limited data on long-term clinical performance [25]
Antibacterial and biocompatible properties [23]	Technique-sensitive application [20]

Despite their numerous advantages, bioactive materials face challenges such as higher costs, technique sensitivity, and limited long-term data [22]. Ongoing research aims to enhance their bioactivity, mechanical properties, and ease of application, ensuring broader adoption in pediatric dentistry [22].

Future advancements in bioactive materials are expected to focus on improving their regenerative potential, incorporating nanotechnology, and enhancing antibacterial properties [22]. These innovations will further reinforce their role in minimally invasive pediatric dentistry [20].

Laser-Assisted Therapies

Laser-assisted technologies have emerged as an integral component of minimally invasive pediatric dentistry, offering effective alternatives to conventional methods for managing both soft and hard tissues. Lasers operate by amplifying light energy, enabling precise tissue removal while minimizing damage to surrounding areas. Their versatile applications include caries removal, cavity preparation, gingivectomy, and pulp therapy, making them highly suitable for pediatric patients who may experience dental anxiety. One of the key advantages of laser systems is their ability to provide pain-free treatments, often eliminating the need for anesthesia, which helps reduce fear and discomfort in children. Additionally, lasers promote hemostasis and accelerate healing, making them ideal for surgical procedures by minimizing bleeding and swelling. Their high precision further supports the principles of MID by preserving healthy tooth structures and reducing the need for extensive tissue removal. However, despite these benefits, several challenges hinder their widespread adoption. High equipment costs, the need for specialized training, and limited availability in low-resource settings remain significant barriers. Ongoing research is focused on developing more affordable laser systems and improving accessibility, which could expand their use in pediatric dentistry while maintaining their effectiveness and patient-friendly nature [24]. Table 6 outlines the benefits and limitations of laser-assisted therapies in dental treatments.

**Table 6:** Benefits and limitations of laser-assisted therapies

Benefits	Limitations
Pain-free and anesthesia-free	High equipment cost
Reduces bleeding and swelling	Requires specialized training
Faster healing and precision	Limited availability in rural areas

3D Printing in Pediatric Dentistry

Three-dimensional (3D) printing has revolutionized minimally invasive pediatric dentistry by enabling the creation of precise, patient-specific restorations, surgical guides, and prostheses. Computer-aided design (CAD)/computer-aided manufacturing (CAM) technology allows clinicians to fabricate restorations using biocompatible materials that ensure durability and esthetics [25]. This advancement has significantly improved treatment planning and execution, allowing for more accurate and efficient dental procedures tailored to pediatric patients.

The applications of 3D printing in pediatric dentistry are diverse, including the production of esthetic crowns and inlays designed to match a child’s dental anatomy, ensuring optimal fit and function. It also facilitates the creation of customized surgical guides that enhance precision during minimally invasive procedures. In orthodontics, 3D printing supports preventive and interceptive treatments through the fabrication of aligners and space maintainers, enabling early intervention and promoting better clinical outcomes [25].

One of the primary benefits of 3D printing is its ability to customize patient-specific designs, improving treatment precision and overall outcomes. Faster production and fitting reduce chair time, which is particularly advantageous for pediatric patients, enhancing compliance and comfort. Additionally, 3D printing enables minimally invasive fabrication techniques, preserving healthy dental structures while delivering functional and esthetic restorations that align with the principles of MID [25].

Despite its numerous advantages, integrating 3D printing technology into clinical practice faces challenges. High costs, reliance on digital infrastructure, and the need for specialized training pose barriers to widespread adoption. Future developments should focus on advancing cost-effective solutions and simplifying workflows to make this technology more accessible in pediatric dentistry. Addressing these limitations will be essential to ensure broader implementation and further enhance the role of 3D printing in delivering high-quality, minimally invasive dental care for children [25]. Table 7 highlights the benefits and limitations of 3D printing technology in dentistry.

**Table 7:** Benefits and limitations of 3D printing in dentistry

Benefits	Limitations
Customizable and precise designs	High initial costs
Reduces chair time for procedures	Digital infrastructure requirements
Enhances esthetics and patient comfort	Training needs for effective use

Advances in Diagnostic Tools for Pediatric Dentistry

Advances in diagnostic technologies have significantly enhanced the early detection and management of dental conditions, supporting minimally invasive approaches in pediatric dentistry. Modern diagnostic tools, such as quantitative light-induced fluorescence (QLF) and near-infrared imaging (NIRI), enable early identification of enamel demineralization and carious lesions. These noninvasive technologies minimize radiation exposure and improve detection accuracy, allowing for timely interventions to prevent lesion progression [26].

Digital imaging systems, including cone-beam computed tomography (CBCT) and optical coherence tomography (OCT), provide high-resolution images that enhance diagnostic precision. CBCT is particularly useful for complex cases involving impacted teeth and root anomalies, while OCT enables detailed visualization of tooth microstructures. These technologies not only aid in diagnosis but also assist in treatment planning and monitoring progress [27].

While these diagnostic advancements improve accuracy and outcomes, they also present challenges. High costs, the need for specialized training, and dependence on digital infrastructure limit their accessibility, particularly in under-resourced areas [27]. Future research aims to simplify these technologies and reduce costs to promote wider adoption. Innovations in AI and machine learning are also being explored to automate diagnosis and enhance clinical decision-making processes [26].

Microdentistry and Nanotechnology in Pediatric Dentistry

Microdentistry and nanotechnology have emerged as transformative approaches in pediatric dentistry, offering precise and minimally invasive solutions for diagnosis, prevention, and treatment. Microdentistry focuses on early diagnosis and targeted interventions, often utilizing magnification tools such as dental microscopes and loupes to detect carious lesions at the earliest stages. These tools enable clinicians to preserve healthy tooth structures while addressing areas of decay effectively [28].

Nanotechnology has further expanded the scope of MID by introducing materials with enhanced mechanical and antibacterial properties. Nanocomposites, for instance, contain nanoparticles that improve strength, wear resistance, and esthetics. These materials provide better adhesion to tooth structures, reducing the risk of secondary caries and enhancing the longevity of restorations. Additionally, nanoparticles such as silver and zinc oxide offer antimicrobial effects, minimizing bacterial growth and supporting oral health [28]

Applications of nanotechnology also extend to preventive treatments, including remineralization agents and dental sealants. Nanohydroxyapatite, a key component of enamel, has shown potential in repairing early-stage carious lesions and strengthening enamel through targeted delivery systems. Such advancements align with the philosophy of MID by focusing on prevention and early intervention [29].

Despite these promising developments, challenges such as high production costs and the need for specialized training continue to limit widespread adoption. Future efforts aim to enhance the affordability and accessibility of nanotechnology-based materials and devices, ensuring their integration into routine pediatric dental practice [28].

AI in Pediatric MID

AI has emerged as a transformative tool in pediatric dentistry, offering advancements in diagnostics, treatment planning, and patient management. AI systems utilize machine learning algorithms to analyze complex dental data, including radiographs, photographs, and clinical records, to detect early signs of dental diseases such as caries, developmental anomalies, and periodontal conditions. These systems demonstrate high accuracy in caries detection and prediction, enabling early interventions that align with minimally invasive principles [30].

AI-based diagnostic tools have proven particularly effective in pediatric dentistry for analyzing radiographic images to identify lesions, supernumerary teeth, and growth abnormalities. Recent studies report AI achieving diagnostic accuracy rates between 87% and 100%, making it a reliable adjunct to traditional techniques [31]. These tools assist clinicians in reducing human error and ensuring consistency, particularly when treating young, uncooperative patients.

In addition to diagnostics, AI aids in treatment planning by simulating outcomes and customizing therapies. For example, AI algorithms assess anatomical variations and treatment histories to predict prognosis, enabling clinicians to design minimally invasive, patient-specific plans. AI also improves workflow efficiency by automating administrative tasks such as scheduling, billing, and patient follow-ups, freeing up time for clinical care [32].

Patient education and behavior management have also benefited from AI-driven applications. Virtual assistants and interactive tools provide educational resources tailored to children, promoting better oral hygiene practices and reducing dental anxiety [33]. These applications ensure a more engaging and supportive dental experience for pediatric patients.

Despite its advantages, integrating AI into pediatric dentistry faces challenges, including data security concerns, ethical considerations, and the need for standardization. Moreover, AI algorithms depend on high-quality datasets, and discrepancies in data collection may affect reliability. Efforts to address these issues focus on improving algorithm transparency, refining training datasets, and enhancing user accessibility [31].

Future developments in AI promise further integration with minimally invasive techniques, enabling real-time monitoring, robotics, and precision treatments. As AI technology evolves, its potential to optimize pediatric dental care while reducing invasiveness and improving outcomes continues to grow [34].

Limitations of minimally invasive techniques in pediatric dentistry

MID offers numerous advantages; however, several limitations may hinder its widespread adoption and implementation in pediatric dental care. High costs associated with advanced technologies like laser systems and 3D printing often restrict accessibility, particularly in low-resource settings. Additionally, techniques such as laser therapy and CAD/CAM systems require specialized training, posing a learning curve for practitioners.

Certain MID procedures, including resin infiltration and ART, are technique-sensitive, demanding precise moisture control and operator skill challenges that may be amplified when managing young and uncooperative patients. While bioactive materials provide effective treatment options, their higher costs and limited long-term clinical data raise concerns regarding durability and performance. Furthermore, esthetic issues, such as discoloration caused by SDF, may reduce acceptance, especially for anterior teeth.

Infrastructure needs, including digital imaging and 3D printing systems, necessitate robust technological support, which may be lacking in rural or underdeveloped areas. Some techniques, like ART and SDF, also require periodic reapplications to maintain efficacy, increasing maintenance costs and time commitments. Addressing these challenges through cost reduction, enhanced training programs, and ongoing research into materials and technologies is essential for improving accessibility and ensuring better clinical outcomes in pediatric dentistry.

## Conclusions

MID is revolutionizing pediatric dental care by prioritizing the preservation of healthy tooth structures, reducing pain, and simplifying treatment procedures. Techniques such as SDF, resin infiltration, ART, laser therapies, and 3D printing enhance patient compliance, clinical efficiency, and long-term outcomes. Advances in bioactive materials, AI, and computer-aided technologies further enable precise and personalized care, expanding MID’s potential.

Despite its promise, broader adoption of MID faces challenges related to cost, training, and infrastructure, particularly in resource-limited settings. Future research should focus on developing affordable materials, improving durability, and integrating AI-driven diagnostics to enhance accessibility. With continued innovations, MID is well-positioned to become the gold standard in pediatric dentistry, ensuring sustainable and patient-centered oral health care globally.
